# Genetic Variants of lncRNA *MALAT1* Exert Diverse Impacts on the Risk and Clinicopathologic Characteristics of Patients with Hepatocellular Carcinoma

**DOI:** 10.3390/jcm8091406

**Published:** 2019-09-06

**Authors:** Lan-Ting Yuan, Jer-Hwa Chang, Hsiang-Lin Lee, Yi-Chieh Yang, Shih-Chi Su, Chien-Liang Lin, Shun-Fa Yang, Ming-Hsien Chien

**Affiliations:** 1 Division of Hepatology and Gastroenterology, Department of Internal Medicine, Yuan’s General Hospital, Kaohsiung 80249, Taiwan; 2 Department of Internal Medicine, School of Medicine, College of Medicine, Taipei Medical University, Taipei 11031, Taiwan; 3 Pulmonary Research Center, Wan Fang Hospital, Taipei Medical University, Taipei 11696, Taiwan; 4 School of Medicine, Chung Shan Medical University, Taichung 40201, Taiwan; 5 Department of Surgery, Chung Shan Medical University Hospital, Taichung 40201, Taiwan; 6 Graduate Institute of Clinical Medicine, College of Medicine, Taipei Medical University, Taipei 11031, Taiwan; 7 Whole-Genome Research Core Laboratory of Human Diseases, Chang Gung Memorial Hospital, Keelung 20401, Taiwan; 8 Department of Pharmacy, FooYin University Hospital, Pingtung 92847, Taiwan; 9 Institute of Medicine, Chung Shan Medical University, Taichung 40201, Taiwan; 10 Department of Medical Research, Chung Shan Medical University Hospital, Taichung 40201, Taiwan; 11 TMU Research Center of Cancer Translational Medicine, Taipei Medical University, Taipei 11031, Taiwan

**Keywords:** long noncoding RNA, metastasis-associated lung adenocarcinoma transcript 1, single nucleotide polymorphisms, susceptibility, clinicopathologic characteristics, hepatocellular carcinoma

## Abstract

The long noncoding (lnc)RNA, *metastasis-associated lung adenocarcinoma transcript 1* (*MALAT1*), plays a crucial role in the development of hepatocellular carcinoma (HCC). However, potential genetic variants (single nucleotide polymorphisms, SNPs) in *MALAT1* that affect the susceptibility and progression of HCC have rarely been explored. Three tagging SNPs, viz., rs3200401 C > T, rs619586 A > G, and rs1194338 C > A, in *MALAT1* were genotyped by a TaqMan allelic discrimination assay in 394 HCC patients and 1199 healthy controls. A stratified analysis showed that younger patients (<55 years) with the *MALAT1* rs619586 G allele had a decreased risk of HCC under a codominant model (AOR = 0.289, 95% CI: 0.108–0.773, *p* = 0.013) and dominant model (AOR = 0.286, 95% CI: 0.107–0.765, *p* = 0.013). Female patients and patients with a smoking habit who carried the CA + AA genotype of rs1194338 had a lower risk of developing vascular invasion (*p* = 0.049) and a high Child–Pugh grade (B or C) (*p* = 0.036), respectively. Under the dominant model, smokers with the *MALAT1* rs3200401 CT + TT genotype had a higher frequency of hepatitis B virus (HBV) infection (*p* = 0.034). Moreover, the aspartate aminotransferase was higher in patients with the rs3200401 CT + TT genotype. Furthermore, analyses of clinical datasets revealed that *MALAT1* expression level was gradually unregulated during HCC development from normal liver, cirrhotic liver, dysplastic liver to HCC and correlated with poor survival rates in HCC patients, especially in the hepatitis virus-infected population.

## 1. Introduction

Liver cancer is the sixth most common malignant tumor with more than 840,000 new cases reported per year worldwide, and this disease is the fourth leading cause of cancer-related deaths [1]. Hepatocellular carcinoma (HCC) is the major type of primary liver cancer, comprising 75–85% of cases. Early-stage HCC can be effectively treated by liver transplantation or curative surgery. However, therapeutic strategies are still limited for advanced disease [2]. The incurable property of HCC is due to its extensive heterogeneity of clinical manifestations, and these heterogeneities are manifested through diverse genetic and epigenetic features, as well as ethnic differences in patients [3,4]. For example, HCC causes higher mortality in developing countries, such as Asian and African countries, because of chronic infection with hepatitis B virus (HBV) or hepatitis C virus (HCV) [1]. In addition, a variety of genetic and epigenetic variations were also reported to be correlated with HCC [5]. Intensive investigations over the last few decades have focused on the roles of protein-coding genes in the pathogenesis of HCC. Recently, research hotspots regarding genetic and epigenetic variations have focused on noncoding (nc)RNAs.

Long noncoding (lnc)RNAs are a type of ncRNA with a length of more than 200 bp, which were identified as oncogenes or tumor suppressors and participate in most processes of tumorigenesis, including uncontrollable proliferation, evasion of cell death, and metastasis [6]. The highly conserved *metastasis-associated lung adenocarcinoma transcript 1* (*MALAT1*) lncRNA with >8000 bp was first identified as an oncogene in non-small-cell lung cancer (NSCLC) because of its functional role in promoting metastasis via induction of the epithelial–mesenchymal transition (EMT) and its clinical significance in predicting metastasis of early-stage NSCLC [7,8]. Subsequently, overexpression of *MALAT1* was shown to exhibit marked effects on tumor cell proliferation, migration, invasion, and apoptosis in numerous types of cancer. Moreover, accumulating evidence indicated that expression levels of *MALAT1* in tumor tissues and/or body fluids could serve as a biomarker for tumor diagnosis and prognosis [9,10]. For instance, aberrant expression of *MALAT1* in colorectal, pancreatic, papillary thyroid, and renal cancers was identified as an unfavorable predictor for their clinical progression and prognosis [11,12,13,14].

Regarding the role of *MALAT1* in HCC, overexpression of *MALAT1* in tumor tissues or sera may correlate with advanced tumor stages and reduced overall survival of HCC patients and indicate a higher risk of tumor recurrence following liver transplantation [15,16]. Moreover, upregulation of *MALAT1* in HCC was reported to be associated with chemoresistance to multiple agents, including 5-fluorouracil, mitomycin C, and Adriamycin via a hypoxia-inducible factor (HIF)-1α-MALAT1-microRNA (mir)-216b pathway [17]. Also, Malakar et al. demonstrated that *MALAT1* enhances HCC progression by inducing serine/arginine-rich splicing factor 1 (SRSF1) upregulation and mammalian target of rapamycin (mTOR) activation [18]. Undoubtedly, *MALAT1* is recognized as an oncogene in HCC.

Results from a whole-genome mutational analysis of an Asian population showed that several mutational signatures were related to liver carcinogenesis including *MALAT1* lncRNA, but their clinical significance remains unclear [19]. Genetic variations such as single-nucleotide polymorphisms (SNPs) can alter expressions of coding genes and lncRNAs. Actually, *MALAT1* SNPs were reported to be associated with the risk or progression of several cancer types and expression of *MALAT1*. For example, the AG genotype or G allele of *MALAT1* rs619586 SNP was reported to be associated with decreased risks of breast cancer, colorectal cancer (CRC), and papillary thyroid cancer (PTC) and a decreased expression level of *MALAT1* [20,21,22]. Moreover, an A allele of rs1194338 in the promoter region of *MALAT1* was also reported to be correlated with a decreased CRC risk [21,23]. Furthermore, the polymorphism rs3200401 C > T in the *MALAT1* gene was recently reported to be associated with an increased risk of esophageal squamous cell carcinoma (ESCC) in a Chinese population [24]. To date, whether polymorphisms of *MALAT1* play any role in the risk and progression of HCC remains less investigated, especially in Asian populations. Accordingly, we performed a case-control study in a Taiwanese population to identify the roles of *MALAT1* SNPs in the risk and clinical characteristics of HCC.

## 2. Materials and Methods

### 2.1. Study Subjects, Ethics, and Consent

HCC patients of this case-control study were recruited from 2007 to 2015 at Chung Shan Medical University Hospital (Taichung, Taiwan), and were composed of 273 men and 121 women. In total, 1199 age- and gender-matched cancer-free controls were randomly selected from the Taiwan Biobank Project, and all study participants were of a Han Chinese population from the same geographic area. In addition to HCC, any patients with a history of other cancer types were excluded. HCC patients were clinically staged at the time of diagnosis according to the tumor, node, metastasis (TNM) staging system of the American Joint Committee on Cancer (AJCC). Demographic data and related risk factors including the smoking and drinking status were obtained through a structured questionnaire. Tobacco consumption was categorized in “never-user” and “ever-user” (current and former user) at the time of diagnosis. Medical information of our recruited HCC patients such as alpha-fetoprotein (AFP), aspartate aminotransferase (AST), alanine aminotransferase (ALT), the TNM status, Child–Pugh grade, vascular invasion, hepatitis virus infection, and liver cirrhosis was obtained from their medical records. Peripheral blood was collected from each participant after informed consent. The research design was approved by the Institutional Review Board of Chung Shan Medical University Hospital.

### 2.2. Genomic DNA Extraction

Whole-blood samples collected from our recruited subjects were placed in tubes containing ethylenediaminetetraacetic acid (EDTA) and further centrifuged for 10 min at 3000 rpm. Buffy coats of DNA were extracted from whole-blood specimens using a QIAamp DNA Blood Mini Kit (Qiagen, Valencia, CA, USA) according to the manufacturer’s instructions. Extracted DNA was dissolved in TE buffer (10 mM Tris and 1 mM EDTA; pH 7.8) and the quality of DNA was evaluated based on a corresponding analysis on a Nanodrop-2000 spectrophotometer (Thermo Scientific, Waltham, MA, USA). The final preparation was stored at −20 °C and was used as templates for the polymerase chain reaction (PCR).

### 2.3. Selection of MALAT1 SNPs

In this study, we selected two tagSNPs, rs3200401 and rs619586, from the International HapMap Project data, because these SNPs fit the following criteria: (i) SNPs located in *MALAT1*; (ii) minor allele frequency (MAF) of each SNP of >5% in a Han Chinese population (rs3200401 C/T: 0.87/0.13; rs619586 A/G: 0.87/0.13; rs1194338 C/A: 0.76/0.24); (iii) linkage disequilibrium value of *r*^2^ < 0.8 for the candidate SNP; and (iv) functional SNPs with a known biological mechanism based on published literature [25,26]. We also included another novel SNP, viz., rs1194338, located in the promoter region of *MALAT1*, which was recently reported to be associated with susceptibility to colorectal carcinoma (CRC) in a Han Chinese population [21].

### 2.4. Genotyping of MALAT1 SNPs

Genotyping of *MALAT1* SNPs, including rs3200401 (assay ID: C_3246069_10), rs619586 (assay ID: C_1060479_10), and rs1194338 (assay ID: C_11661801_10), was done using the TaqMan SNP Genotyping Assay with an ABI StepOnePlus™ Real-Time PCR System and further evaluated with SDS version 3.0 software (Applied Biosystems, Foster City, CA, USA).

### 2.5. Bioinformatics Analysis

The Genome-Tissue Expression (GTEx) database (https://www.gtexportal.org/home/) [27] was used to identify the regulatory potential of candidate functional *MALAT1* variants. GTEx data were used to identify correlations between SNPs and tissue-specific gene expression levels. Transcriptomic data from samples of a normal liver, cirrhotic liver, dysplastic liver, and HCC were analyzed using the microarray datasets with accession number GSE6764 obtained from the Gene Expression Omnibus (GEO) database (https://www.ncbi.nlm.nih.gov/geo/) to investigate whether *MALAT1* expression (probe ID: 224568_x_at) was associated with HCC tumor formation. RNA sequencing analysis, the visualization platform Gene Expression Profiling Interactive Analysis (GEPIA) (http://gepia.cancer-pku.cn/) [28], and the Kaplan–Meier (KM) plotter (http://kmplot.com/analysis/) [29] were used to determine whether *MALAT1* expression was correlated with the prognosis of HCC patients. The correlation of *MALAT1* and prognosis were calculated by median cutoff in GEPIA and auto selected best cutoff in KM-plotter analysis. The GEPIA performs data mining based on The Cancer Genome Atlas (TCGA) data.

### 2.6. Statistical Analysis

To compare age, gender, demographic characteristic distributions and categorical variables between HCC patients and control groups, the Mann–Whitney *U*-test and Chi-squared test was applied for statistical analysis. Multiple logistic regression models were used to estimate the adjusted odds ratio (AOR) and 95% confidence interval (CI) of the association of genotype frequencies with HCC risk; *p* < 0.05 was considered statistically significant. Experimental data were analyzed using SAS statistical software (vers. 9.1, 2005; SAS Institute, Cary, NC, USA).

## 3. Results

### 3.1. Demographic Characteristics of Recruited Subjects

Distributions of demographic and clinical characteristics of recruited subjects are shown in Table 1. No significant differences were found between HCC patients and the controls with regard to the distribution of age <55 and ≥55 years (*p* = 0.451), gender (*p* = 0.797), or smoking status (*p* = 0.913). In contrast, significantly higher frequencies of HCC patients compared to controls had a drinking habit (14.1% vs. 35.3%; *p* < 0.001) and were positive for hepatitis B surface antigen (HBsAg) (12.2% vs. 42.4%; *p* < 0.001) and anti-HCV antibodies (4.4% vs. 45.2%; *p* < 0.001). Among HCC patients, higher proportions were diagnosed as being at the early clinical (I + II) and T (T1 + T2) stages, and with no lymph node (N0) or distal (M0) metastasis. Most patients had slight liver cirrhosis (Child–Pugh grade 0 or A).

### 3.2. Associations between MALAT1 Candidate SNPs and HCC Susceptibility

To examine possible associations of *MALAT1* SNPs with the risk of developing HCC, the genotype frequencies of three SNPs (rs3200401, rs619586, and rs1194338) were first investigated in the entire population we recruited. Alleles with the highest distribution frequencies for rs3200401, rs619586, and rs1194338 SNPs of our recruited subjects were homozygous C/C, A/A, and C/C, respectively. Genotypic distributions of *MALAT1* rs3200401, rs619586, and rs1194338 in the control group conformed to Hardy–Weinberg equilibrium (*p* = 0.112, χ^2^ value: 2.521; *p* = 0.423, χ^2^ value: 0.641 and *p* = 0.566, χ^2^ value: 0.330, respectively). After adjusting for age, gender, cigarette smoking, and alcohol consumption, no significant differences were observed between HCC patients with these candidate SNPs (rs3200401 CT/TT, rs619586 AG/GG, rs1194338 CA/AA) and those with the wild-type (WT) gene (Table 2). We further divided the tested population by age, and found that younger patients (<55 years) with the AG/GG genotypes of *MALAT1* rs619586 had a decreased risk of HCC under the codominant model (AG vs. AA: AOR, 0.289; 95% CI, 0.108–0.773; *p* = 0.013) and dominant model (AG + GG vs. AA: AOR, 0.286; 95% CI, 0.107–0.765; *p* = 0.013) (Table 3), while no associations were observed in elderly HCC patients (≥55 years) (data not shown).

### 3.3. Relationships between Clinicopathological Features of HCC and MALAT1 SNPs in Different Genders

Next, to clarify the effects of *MALAT1* genetic polymorphisms on the HCC clinicopathological status, including clinical stage, primary tumor size, lymph node and distant metastasis, vascular invasion, Child–Pugh grade, hepatitis virus infection, and liver cirrhosis. Among all HCC patients, the rs3200401, rs619586, and rs1194338 SNPs showed no significant association with the clinicopathologic status (Table S1). We further divided the HCC population by sex, and found that only the female HCC patients who carried at least one minor allele (CA and AA) of rs1194338 had a significantly (*p* = 0.049) 0.377-fold lower risk (95% CI, 0.143–0.994) of developing vascular invasion compared to their corresponding WT homozygotes (Table 4 and Table S2).

### 3.4. Relationships between Clinicopathological Features and MALAT1 SNPs in HCC Patients Who Smoked Tobacco

We further divided HCC patients into smoking and nonsmoking subgroups and investigated differences between *MALAT1* SNPs and the HCC clinicopathological status in these two groups. No association between *MALAT1* SNPs (rs3200401, rs619586, and rs1194338) and HCC clinical features was observed in nonsmoking subgroup (Table S3). Among 156 HCC smokers, we observed that patients with at least one polymorphic A allele at the rs1194338 SNP (CA + AA genotype) had lower risks of developing moderate to severe liver failure (Child–Pugh B or C grade; *p* = 0.036) (Table 5 and Table S3). Moreover, patients with at least one polymorphic T allele at the rs3200401 SNP (CT + TT genotype) had a higher risk of being infected with HBV (*p* = 0.034) than did WT patients (Table 6 and Table S3).

### 3.5. Serum Alpha-Fetoprotein (AFP), Aspartate Aminotransferase (AST), and Alanine Aminotransferase (ALT) Levels among Different MALAT1 SNP Carriers in HCC Patients

Serum AFP is a common clinical pathological marker of HCC. Serum ALT and AST levels are most often used to evaluate liver function [30]. Table S4 shows associations of MALAT1 genotypic frequencies with the HCC laboratory status. After adjusting for age, gender, cigarette smoking, and alcohol consumption, we observed that AST levels were significantly higher in HCC patients harboring the rs3200401 CT + TT genotype compared to those with CC homozygotes. Otherwise, no significant associations between levels of these HCC clinical pathologic markers and genotypes of any other MALAT1 SNPs (rs619586 and rs1194338) were noted.

### 3.6. Clinical Relevance of MALAT1 Expression Levels in HCC Patients with or without Hepatitis Infection

**Figure 1:**
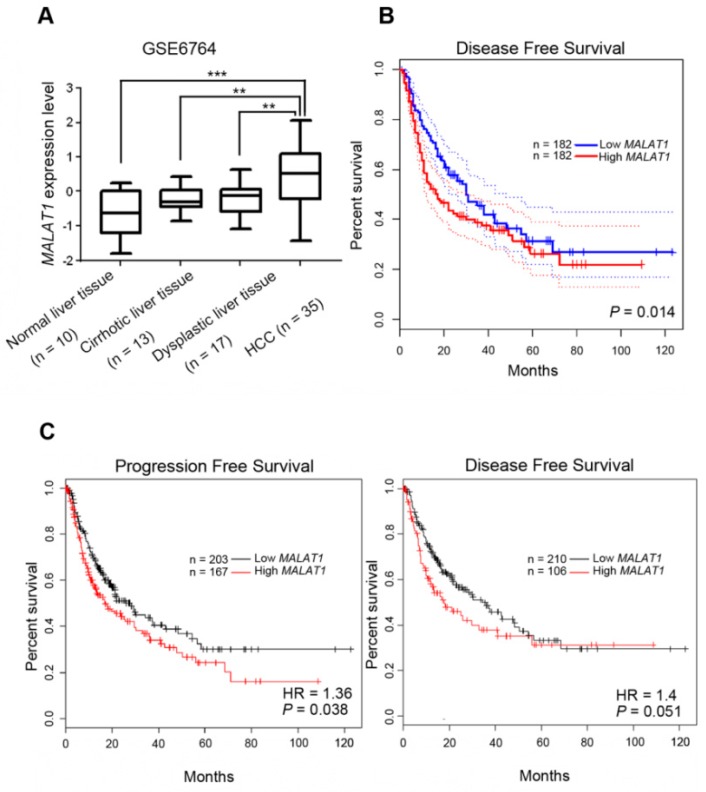
Clinical relevance of MALAT1 expression in the development and prognosis of hepatocellular carcinoma (HCC). (**A**) MALAT1 gene expression levels in normal liver, cirrhotic liver, dysplastic liver, and HCC tissues compared according to data from GEO datasets (GSE6764). (**B**,**C**) Kaplan–Meier plots of disease-free and progression-free survival of HCC patients stratified by MALAT1 expression, which were analyzed by GEPIA (**B**) and KM plotter (**C**), respectively. A log-rank test was used to examine between-group differences. HR, hazard ratio.

**Figure 2:**
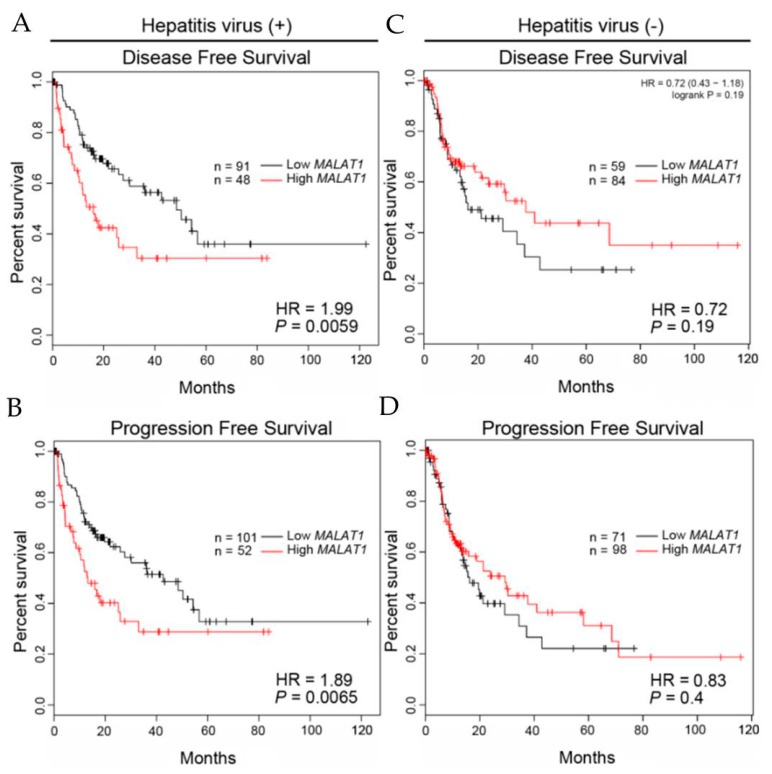
Prognostic significance of *MALAT1* in hepatocellular carcinoma (HCC) patients with (**A**,**B**) or without (**C**,**D**) hepatitis virus infection. Kaplan–Meier plots of disease-free survival (DFS) and progression-free survival (PFS) of HCC patients with or without hepatitis virus infection which were analyzed by the KM plotter. Overall DFS and PFS predicted by low versus high *MALAT1* expression. HR, hazard ratio.

Considering the potential effects of MALAT1 polymorphic genotypes on MALAT1 expression levels, we further clarified the clinical significance of MALAT1 in HCC formation and progression. Tissues of different stages of HCC formation including normal liver, cirrhotic liver, dysplastic liver, and HCC were first analyzed from the GEO database (GSE6764), and we found significantly higher *MALAT1* transcripts in HCC tissues than in normal liver, cirrhotic liver, and dysplastic liver tissues (Figure 1A). *MALAT1* expression in the cirrhotic liver or dysplastic liver also showed higher trends than in the normal liver, suggesting that *MALAT1* might play a role in the process of HCC development. Moreover, *MALAT1* expression data in TCGA database were analyzed using the online tool, GEPIA [28], and we found that HCC patients with *MALAT1*^high^ tumors had shorter disease free survival (DFS) times compared to those with *MALAT1*^low^ tumors (*p* = 0.014; Figure 1B). Furthermore, correlations between *MALAT1* expression and DFS or progression-free survival (PFS) in HCC patients were analyzed using the KM plotter [29], and we found that *MALAT1* expression was negatively correlated with DFS (*p* = 0.051) and the PFS time (*p* = 0.038; Figure 1C). All tested HCC patients from the KM plotter were divided by the presence or absence of hepatitis virus infection, and we found that the negative correlation of *MALAT1* levels with DSF (*p* = 0.0059) or PFS (*p* = 0.0065) time was only observed in the hepatitis virus-infected population, and this correlation was more striking in the virus-infected population than in the overall population (Figure 1C and Figure 2). Taken together, the above clinical data suggest that *MALAT1* expression may modulate the formation and progression of HCC. Hepatitis virus infection may play a critical role in *MALAT1*-modulated HCC progression.

## 4. Discussion

HCC is one of the most prevalent forms of cancer, and its incidence is increasing at a very high rate. To date, the pathophysiology and underlying causes of HCC are less well understood. Recently, accumulating studies have focused on the causative ncRNAs involved in HCC development, which may assist us in understanding the still unclear molecular processes involved in HCC [31,32]. lncRNAs are a type of ncRNA which were reported to exhibit tissue-specific expression patterns and have high utility as prognostic biomarkers for various cancer types [33,34]. Moreover, lncRNAs are expressed at low copy numbers [35] and show high diversity in transcripts in different tissues. Actually, numerous lncRNAs function as oncogenes or tumor-suppressor genes, which were reported to be involved in metastasis and prognosis of HCC, such as *MEG3*, *GAS5*, *HOTAIR*, *HULC*, *H19*, and *MALAT1* [36]. The oncogenic roles of *MALAT1* in HCC were previously extensively studied, such as promotion of motility, proliferation, and metastasis of HCC through inducing latent transforming growth factor β-binding protein 3 (LTBP3) expression and obliterating anticancer effects of miR146-5p and mir-195 [37,38,39]. However, the epidemiological focus on HCC susceptibility and clinicopathologic characteristics conferred by genetic variants on loci of *MALAT1* have rarely been investigated.

The present study demonstrated that individuals younger than 55 years with the mutant base G of rs619586 had a significantly lower risk of HCC, regardless of the codominant model (OR: 0.684, 95%CI: 0.478–0.979) or dominant model (OR: 0.675, 95%CI: 0.479–0.951). Previous studies conducted in Chinese populations also showed that the AG, GG genotype or G allele of *MALAT1* rs619586 polymorphism was significantly associated with a decreased risk of breast cancer, CRC, ESCC, and PTC [20,21,22,24]. Genetic variants of lncRNA were reported to influence its expression or function due to structural changes and further contribute to cancer development [40]. Indeed, miR-214 was reported to directly target *MALAT1* with the rs619586 G allele and further induce downregulation of *MALAT1* [25]. Actually, messenger (m)RNA expression levels of *MALAT1* were significantly lower in PTC patients or cancer-free subjects who harbored the AG + GG genotype than those with the AA genotype [20,22]. Those results suggested that genetic mutations of *MALAT1* rs619586 might affect the corresponding expression of mRNA through miR-214 binding and further affect the development of HCC. Relationships between *MALAT1* rs619586 SNPs and miR-214 to regulate *MALAT1* expression in HCC should be further addressed in the future.

HCC is characterized by its propensity for vascular invasion, and *MALAT1* was reported to promote invasion by HCC cells. In non-small cell lung cancer (NSCLC), higher *MALAT1* expression was significantly related to female sex, TMN stage, vessel invasion, and recurrence [41]. In the current study, a novel genetic variant, viz., rs1194338, in the promoter region of *MALAT1* was evaluated, and we found that female HCC patients with the *MALAT1* rs1194338 A allele were significantly associated with a decreased risk of developing vascular invasion compared to those with the C allele. We also observed that a higher *MALAT1* level was correlated with poor DFS and PFS in patients with HCC based on a bioinformatics analysis. It was reported that genetic variants in the promoter region can affect the expression, stability, and subcellular localization of the transcriptome, resulting in functional changes and disease occurrence [42]. However, Li et al. indicated that the rs1194338 polymorphism (C > A) did not affect expression levels of *MALAT1* in CRC tissues [23]. In contrast to colon tissues, data from the GTEx database [27] showed that a significant decrease of *MALAT1* expression with the rs1194338 SNP (CA + AA) compared to the WT genotype (CC, *p* = 0.000095) was observed in 491 skeletal muscle tissue samples. We hypothesized that the functional relevance of rs1194338 on *MALAT1* expression might be tissue specific, and further studies are needed to investigate the correlation between the rs1194338 SNP and *MALAT1* expression and the precise mechanism underlying the function of rs1194338 SNP in HCC. In addition, sex hormones have been considered to play a role in HCC development. Estrogen is the main female sex hormone. Recently, estrogen was reported to attenuate HCC progression by regulating cell proliferation and invasion [43]. Moreover, high estrogen was shown to suppress invasion of breast cancer cells through decreasing MALAT1 level [44]. However, the correlations among genetic variant rs1194338, estrogen concentration, and vascular invasion of female HCC patients also need to be further investigated.

Tobacco smoking is reported to be associated with HBV-related HCC recurrence and patient survival after curative surgery. More HCC patients with liver function of Child–Pugh class B were identified in current smokers than nonsmokers [45]. *MALAT1* was reported to be involved in hepatitis B virus X protein (HBx)-mediated HCC formation and to be induced by HBx in HCC cells [37]. Actually, we only observed that *MALAT1* expression was a poor prognostic factor in hepatitis virus-infected HCC patients. Moreover, cigarette smoke extract was also reported to induce upregulation of *MALAT1* in human bronchial epithelial cells [46]. In the current study, HCC patients were further divided into smoking and nonsmoking groups, and we further investigated differences between *MALAT1* genetic variants and the HCC clinicopathological status. We found that smokers with the *MALAT1* rs1194338 polymorphism (CA + AA) had a lower risk of developing moderate to severe liver failure (Child–Pugh B or C grade). In addition, smokers with the *MALAT1* rs3200401 polymorphism (CT + TT) had a higher frequency of HBV infection. We speculated that tobacco carcinogens might alter *MALAT1* expression dependent upon the presence of the rs1194338 and rs3200401 polymorphisms and further influence the severity of liver failure and frequency of HBV infection in HCC patients. Furthermore, it was reported that interactions of lncRNAs and other molecules were probably correlated with their structures, which are influenced by genetic variations of lncRNAs [47]. The C > T mutation of rs3200401 may alter the structural features of *MALAT1* and result in loss of *MALAT1*-*SRSF2* binding, downregulation of *SRSF2* phosphorylation, changes in the alternative splicing of pre-mRNAs, and finally changes in expression levels of cancer-associated genes [26]. Whether rs3200401 polymorphism-related *MALAT1*-*SRSF2* binding loss might affect HBV infection in HCC patients needs to be further investigated.

A recent study from Xiaohui Ji et al. investigated the associations between *MALAT1* SNPs and HCC and indicated that three intronic SNPs, rs11227209, rs619586, and rs3200401, at *MALAT1* were not associated with HCC susceptibility and clinicopathological features in a southern Chinese population [48]. They also showed no significant association between *MALAT1 rs619586* and HCC risk in HBV-positive subgroup. Similar to Ji’s study, we also observed that SNPs rs619586 and rs3200401 were not significantly associated with HCC susceptibility and clinicopathological features in a Taiwanese population. HBV and HCV are independent factor of HCC. No matter in HBV- or HCV-infected HCC patients, no significant associations were observed between *MALAT1* rs619586 and rs3200401 and clinicopathologic status in our study (Tables S5 and S6). In addition to intronic SNPs, a novel genetic variant, viz., rs1194338, in the promoter region of *MALAT1* was first evaluated in HCC in our study. Although *MALAT1* rs1194338 SNPs did not correlate with HCC susceptibility either, we found that female HCC patients with the *MALAT1* rs1194338 A allele were significantly associated with a decreased risk of developing vascular invasion compared to those with the C allele. In hepatitis virus-infected HCC patients, we observed that MALAT1 rs1194338 SNPs did not associate with clinicopathologic status. Combined effects of environmental factors and *MALAT1* gene SNPs on the risk of HCC were also analyzed by Ji’s study and showed no significant association between *MALAT1* rs11227209, rs619586, rs3200401 and HCC susceptibility in HCC patients with different environmental factors exposure. In contrast, our study revealed that HCC smokers with the *MALAT1* rs1194338 polymorphism (CA + AA) had a lower risk of developing moderate to severe liver failure. In addition, smokers with the *MALAT1* rs3200401 polymorphism (CT + TT) had a higher frequency of HBV infection. The differences between these association results from Ji’s and our studies might be due to dissimilarities in the genetic background of participants and the tissue-specific effect of lncRNA.

Our study is not without limitations. First, the sample size of the current study was still not large enough and might lead to a limited statistical impact on the accuracy and precision of the results. For example, in some statistical analyses of selected subgroups (e.g., vascular invasive status of female population), the rather small number of patients might mean that the statistical significance was practically irrelevant (*p* = 0.049). Moreover, although the clinical data from Figure 2 suggested that hepatitis virus infection may influence MALAT1-modulated HCC progression. The rather small number of hepatitis virus-infected patients might cause the insignificant associations between *MALAT1* SNPs and clinicopathologic status in hepatitis virus-infected HCC patients. Therefore, larger sample sizes from other medical centers are needed to further confirm the role of *MALAT1* SNPs in HCC susceptibility and development, especially in HBV- or HCV-infected populations. Moreover, this study is restricted to the Taiwanese population; the effect of *MALAT1* SNPs on HCC risk in other ethnic populations is necessary to compare with and double-check our current results. Furthermore, in our assessment of *MALAT1* association with clinical parameters of HCC, we have performed a multivariate analysis to control potential confounding factors. However, another weakness is that we did not adjust for multiple testing to avoid type I error. The association detected in the present study, therefore, might be limited unless additional replication experiments are conducted. Finally, the functional relevance of *MALAT1* SNPs in HCC is still unclear, the mRNA and DNA should be collected simultaneously from the same samples to further validate the effect of *MALAT1* SNPs on *MALAT1* expression in future studies.

## 5. Conclusions

Despite our best efforts, a significant proportion of HCC patients will eventually develop advanced disease, and we do not currently have reliable tools to predict who those patients are. In this study, we identified the diverse allelic effects of *MALAT1* genetic variants (rs3200401, rs619586, and rs1194338) which contribute to the susceptibility and clinicopathologic development of HCC in a Taiwanese population. These results can lead to a better understanding of risk and early detection of HCC.

## Figures and Tables

**Table 1:** Distributions of demographic characteristics in 1199 controls and 394 patients with hepatocellular carcinoma.

Variable	Controls (*N* = 1199) *n* (%)	Patients (*N* = 394) *n* (%)	*p* Value
Age (years)			
≥55	912 (76.1%)	307 (77.9%)	0.451
Gender			
Male	839 (70%)	273 (69.3%)	0.797
Cigarette smoking			
Yes	471 (39.3%)	156 (39.6%)	0.913
Alcohol consumption			
Yes	169 (14.1%)	139 (35.3%)	<0.001 *
HBsAg			
Positive	146 (12.2%)	167 (42.4%)	<0.001 *
Anti-HCV			
Positive	53 (4.4%)	178 (45.2%)	<0.001 *
AFP (ng/mL) ^a^		20.83	
AST (IU/L) ^a^		48	
ALT (IU/L) ^a^		43	
Stage			
III + IV		117 (29.7%)	
Tumor T status			
T3 + T4		112 (28.4%)	
Lymph node status			
N1 + N2 + N3		12 (3%)	
Metastasis			
M1		19 (4.8%)	
Child–Pugh grade			
B or C		74 (18.8%)	
Liver cirrhosis			
Positive		326 (82.7%)	

Mann–Whitney U-test or Chi-squared test was used between healthy controls and patients with HCC. * *p* value < 0.05 as statistically significant. ^a^ Median level. HBsAg, hepatitis B surface antigen; HCV, hepatitis C virus; AFP, alpha-fetoprotein; AST, aspartate aminotransferase; ALT, alanine aminotransferase.

**Table 2:** Genotyping and allele frequency of *MALAT1* single nucleotide polymorphisms (SNPs) in hepatocellular carcinoma and normal controls.

Variable	Controls (*N* = 1199) *n* (%)	Patients (*N* = 394) *n* (%)	OR (95% CI)	AOR (95% CI) ^a^
rs3200401				
CC	802 (66.9%)	263 (66.8%)	1.000 (reference)	1.000 (reference)
CT	347 (28.9%)	117 (29.7%)	1.028 (0.799–1.322)	1.051 (0.805–1.372)
TT	50 (4.2%)	14 (3.6%)	0.854 (0.465–1.570)	0.881 (0.459–1.689)
CT + TT	397 (33.1%)	131 (33.2%)	1.006 (0.790–1.281)	1.030 (0.797–1.331)
rs619586				
AA	1014 (84.6%)	330 (83.8%)	1.000 (reference)	1.000 (reference)
AG	175 (14.6%)	61 (15.5%)	1.071 (0.780–1.470)	0.982 (0.701–1.376)
GG	10 (0.8%)	3 (0.8%)	0.922 (0.252–3.370)	0.563 (0.141–2.250)
AG + GG	185 (15.4%)	64 (16.2%)	1.063 (0.780–1.450)	0.954 (0.686–1.327)
rs1194338				
CC	510 (42.5%)	172 (43.7%)	1.000 (reference)	1.000 (reference)
CA	537 (44.8%)	175 (44.4%)	0.966 (0.758–1.232)	0.980 (0.759–1.266)
AA	152 (12.7%)	47 (11.9%)	0.917 (0.633–1.327)	0.995 (0.672–1.474)
CA + AA	689 (57.5%)	222 (56.3%)	0.955 (0.759–1.202)	0.983 (0.772–1.253)

The odds ratios (ORs) with their 95% confidence intervals (CIs) were estimated by logistic regression models. AOR, adjusted odds ratio. ^a^ Adjusted for the effects of age, gender, cigarette smoking and alcohol consumption.

**Table 3:** Genotyping and allele frequency of *MALAT1* single nucleotide polymorphisms (SNPs) in hepatocellular carcinoma and normal controls among those aged <55 years.

Variable	Controls (*N* = 287) *n* (%)	Patients (*N* = 87) *n* (%)	OR (95% CI)	AOR (95% CI) ^a^
rs3200401				
CC	193 (67.2%)	55 (63.2%)	1.000 (reference)	1.000 (reference)
CT	82 (28.6%)	30 (34.5%)	1.284 (0.768–2.147)	1.452 (0.837–2.516)
TT	12 (4.2%)	2 (2.3%)	0.585 (0.127–2.692)	0.725 (0.148–3.548)
CT + TT	94 (32.8%)	32 (36.8%)	1.195 (0.724–1.971)	1.364 (0.799–2.330)
rs619586				
AA	239 (83.3%)	82 (94.3%)	1.000 (reference)	1.000 (reference)
AG	47 (16.4%)	5 (5.7%)	0.310 (0.119–0.806) *p* = 0.016 *	0.289 (0.108–0.773) *p* = 0.013 *
GG	1 (0.3%)	0 (0%)	NA	NA
AG + GG	48 (16.7%)	5 (5.7%)	0.304 (0.117–0.789) *p* = 0.014 *	0.286 (0.107–0.765) *p* = 0.013 *
rs1194338				
CC	124 (43.2%)	36 (41.4%)	1.000 (reference)	1.000 (reference)
CA	126 (43.9%)	42 (48.3%)	1.148 (0.690–1.911)	1.313 (0.760–2.266)
AA	37 (12.9%)	9 (10.3%)	0.838 (0.370–1.898)	1.330 (0.548–3.232)
CA + AA	163 (56.8%)	51 (58.6%)	1.078 (0.663–1.753)	1.316 (0.778–2.224)

The odds ratios (ORs) with their 95% confidence intervals (CIs) were estimated by logistic regression models. ^a^ Adjusted for the effects of gender, cigarette smoking, and alcohol consumption. * *p* value < 0.05 as statistically significant. NA, not applicable; AOR, adjusted odds ratio.

**Table 4:** Odds ratio (OR) and 95% confidence interval (CI) of clinical status and *MALAT1* rs1194338 genotypic frequencies in 121 female hepatocellular carcinoma patients.

Variable	Genotypic Frequencies			
	CC (%) (*n* = 51)	CA + AA (%) (*n* = 70)	OR (95% CI)	*p* Value
Clinical Stage				
Stage III/IV	12 (23.5%)	21 (30%)	1.393 (0.611–3.177)	0.431
Tumor size				
>T2	9 (17.6%)	20 (28.6%)	1.867 (0.769–4.533)	0.168
Lymph node metastasis				
Yes	2 (3.9%)	0 (0%)	NA	NA
Distant metastasis				
Yes	3 (5.9%)	2 (2.9%)	0.471 (0.076–2.925)	0.419
Vascular invasion				
Yes	13 (25.5%)	8 (11.4%)	0.377 (0.143–0.994)	0.049 *
Child–Pugh grade				
B or C	12 (23.5%)	13 (18.6%)	0.741 (0.306–1.794)	0.507
HBsAg				
Positive	16 (31.4%)	22 (31.4%)	1.003 (0.461–2.182)	0.995
Anti-HCV				
Positive	28 (54.9%)	39 (55.7%)	1.033 (0.500–2.135)	0.929
Liver cirrhosis				
Positive	45 (88.2%)	58 (82.9%)	0.644 (0.225–1.850)	0.414

The ORs with their 95% CIs were estimated by logistic regression models. >T2: multiple tumors of >5 cm or tumors involving a major branch of the portal or hepatic vein(s). * *p* value < 0.05 as statistically significant. NA, not applicable; HBsAg, hepatitis B surface antigen; HCV, hepatitis C virus.

**Table 5:** Odds ratio (OR) and 95% confidence interval (CI) of the clinical status and *MALAT1* rs1194338 genotypic frequencies in 156 hepatocellular carcinoma patients among smokers.

Variable	Genotypic Frequencies			
	CC (%) (*n* = 64)	CA + AA (%) (*n* = 92)	OR (95% CI)	*p* Value
Clinical stage				
Stage III/IV	23 (35.9%)	26 (28.3%)	0.702 (0.355–1.39)	0.311
Tumor size				
>T2	23 (35.9%)	26 (28.3%)	0.702 (0.355–1.39)	0.311
Lymph node metastasis				
Yes	2 (3.1%)	3 (3.3%)	1.045 (0.170–6.439)	0.962
Distant metastasis				
Yes	2 (3.1%)	5 (5.4%)	1.782 (0.335–9.482)	0.498
Vascular invasion				
Yes	14 (21.9%)	11 (12%)	0.485 (0.204–1.152)	0.101
Child–Pugh grade				
B or C	17 (26.6%)	12 (13%)	0.415 (0.182–0.944)	0.036 *
HBsAg				
Positive	28 (43.8%)	38 (41.3%)	0.905 (0.475–1.725)	0.761
Anti-HCV				
Positive	31 (48.4%)	40 (43.5%)	0.819 (0.432–1.554)	0.541
Liver cirrhosis				
Positive	53 (82.8%)	77 (83.7%)	1.065 (0.454–2.500)	0.884

The ORs with their 95% CIs were estimated by logistic regression models. >T2: multiple tumors of >5 cm or tumors involving a major branch of the portal or hepatic vein(s). * *p* value < 0.05 as statistically significant. HBsAg, hepatitis B surface antigen; HCV, hepatitis C virus.

**Table 6:** Odds ratio (OR) and 95% confidence interval (CI) of clinical status and *MALAT1* rs3200401 genotypic frequencies in hepatocellular carcinoma patients among smokers.

Variable	Genotypic Frequencies			
	CC (%) (*n* = 100)	CT + TT (%) (*n* = 56)	OR (95% CI)	*p* Value
Clinical stage				
Stage III/IV	34 (34%)	15 (26.8%)	0.710 (0.345–1.462)	0.353
Tumor size				
>T2	34 (34%)	15 (26.8%)	0.710 (0.345–1.462)	0.353
Lymph node metastasis				
Yes	5 (5%)	0 (0%)	NA	NA
Distant metastasis				
Yes	6 (6%)	1 (1.8%)	0.285 (0.033–2.428)	0.251
Vascular invasion				
Yes	18 (18%)	7 (12.5%)	0.651 (0.254–1.669)	0.371
Child–Pugh grade				
B or C	19 (19%)	10 (17.9%)	0.927 (0.397–2.162)	0.860
HBsAg				
Positive	36 (36%)	30 (53.6%)	2.051 (1.055–3.990)	0.034 *
Anti-HCV				
Positive	48 (48%)	23 (41.1%)	0.755 (0.390–1.463)	0.405
Liver cirrhosis				
Positive	80 (80%)	50 (89.3%)	2.083 (0.783–5.542)	0.141

The ORs with their 95% CIs were estimated by logistic regression models. >T2: multiple tumors of >5 cm or tumors involving a major branch of the portal or hepatic vein(s). * *p* value < 0.05 as statistically significant. NA, not applicable; HBsAg, hepatitis B surface antigen; HCV, hepatitis C virus.

## Supplementary Materials

The following are available online at https://www.mdpi.com/2077-0383/8/9/1406/s1, Table S1: Odds ratio (OR) and 95% confidence interval (CI) of clinical status and *MALAT1* genotypic frequencies in 394 hepatocellular carcinoma patients, Table S2: Distribution frequency of the clinical status and for *MALAT1* SNPs frequencies in hepatocellular carcinoma patients with stratified by gender, Table S3: Distribution frequency of the clinical status and for *MALAT1* SNPs frequencies in hepatocellular carcinoma patients with stratified by smoking status, Table S4: Association of *MALAT1* genotypic frequencies with hepatocellular carcinoma laboratory findings, Table S5: Odds ratio (OR) and 95% confidence interval (CI) of clinical status and *MALAT1* genotypic frequencies in hepatocellular carcinoma patients with hepatitis B virus infection (*n* = 167), Table S6: Odds ratio (OR) and 95% confidence interval (CI) of clinical status and *MALAT1* genotypic frequencies in hepatocellular carcinoma patients with hepatitis C virus infection (*n* = 178).
