# Supplementary material for: Genetic Variants of lncRNA MALAT1 Exert Diverse Impacts on the Risk and Clinicopathologic Characteristics of Patients with Hepatocellular Carcinoma
Source: J Clin Med. 2019 Sep 6;8(9):1406. doi: 10.3390/jcm8091406 (PMC6780489; doi:10.3390/jcm8091406)
Supplement: Supplementary file 1 [file jcm-08-01406-s001.pdf]

Supplementary data

Table 1. Odds ratio (OR) and 95% confidence interval (CI) of clinical status and *MALAT1* genotypic frequencies in 394 hepatocellular carcinoma patients.

Variable	Genotypic frequencies		
	rs3200401	rs619586	rs1194338
Clinical Stage (Stage I/II vs Stage III/IV)	*p* = 0.497	*p* = 0.766	*p* = 0.494
Tumor size (≦T2 vs >T2)	*p* = 0.596	*p* = 0.807	*p* = 0.515
Lymph node metastasis (No vs Yes)	*p* = 0.541	*p* = 0.461	*p* = 0.888
Distant metastasis (No vs Yes)	*p* = 0.513	*p* = 0.493	*p* = 0.541
Vascular invasion (No vs Yes)	*p* = 0.290	*p* = 0.728	*p* = 0.100
Child-Pugh grade (A vs B or C)	*p* = 0.914	*p* = 0.489	*p* = 0.083
HBsAg (Negative vs Positive)	*p* = 0.162	*p* = 0.388	*p* = 0.295
Anti-HCV (Negative vs Positive)	*p* = 0.639	*p* = 0.600	*p* = 0.885
Liver cirrhosis (Negative vs Positive)	*p* = 0.194	*p* = 0.287	*p* = 0.471

Table 2. Distribution frequency of the clinical status and for *MALAT1* SNPs frequencies in hepatocellular carcinoma patients with stratified by gender.

Variable	Male			Female		
	rs3200401	rs619586	rs1194338	rs3200401	rs619586	rs1194338
Clinical Stage (Stage I/II vs Stage III/IV)	*p* = 0.539	*p* = 0.383	*p* = 0.745	*p* = 0.715	*p* = 0.509	*p* = 0.431
Tumor size (≦T2 vs. > T2)	*p* = 0.603	*p* = 0.352	*p* = 0.955	*p* = 0.770	*p* = 0.415	*p* = 0.168
Lymph node metastasis (No vs Yes)	*p* = 0.746	*NA*	*p* = 0.360	*NA*	*p* = 0.301	*NA*
Distant metastasis (No vs Yes)	*p* = 0.617	*p* = 0.411	*p* = 0.232	*p* = 0.630	*p* = 0.954	*p* = 0.419
Vascular invasion (No vs Yes)	*p* = 0.248	*p* = 0.384	*p* = 0.534	*p* = 0.896	*p* = 0.546	*p* = 0.049*
Child-Pugh grade (A vs B or C)	*p* = 0.728	*p* = 0.247	*p* = 0.096	*p* = 0.444	*p* = 0.668	*p* = 0.507
HBsAg (Negative vs Positive)	*p* = 0.194	*p* = 0.254	*p* = 0.240	*p* = 0.766	*p* = 0.698	*p* = 0.995
Anti-HCV (Negative vs Positive)	*p* = 0.497	*p* = 0.565	*p* = 0.961	*p* = 0.670	*p* = 0.732	*p* = 0.929
Liver cirrhosis (Negative vs Positive)	*p* = 0.431	*p* = 0.278	*p* = 0.715	*p* = 0.198	*p* = 0.707	*p* = 0.414

* *p* value < 0.05 as statistically significant. NA: not applicable.

Table 3. Distribution frequency of the clinical status and for *MALAT1* SNPs frequencies in hepatocellular carcinoma patients with stratified by smoking status.

Variable	Smokers			Non-smokers		
	rs3200401	rs619586	rs1194338	rs3200401	rs619586	rs1194338
Clinical Stage (Stage I/II vs Stage III/IV)	*p* = 0.353	*p* = 0.945	*p* = 0.311	*p* = 0.895	*p* = 0.740	*p* = 0.093
Tumor size (≦T2 vs. >T2)	*p* = 0.353	*p* = 0.945	*p* = 0.311	*p* = 0.963	*p* = 0.788	*p* = 0.101
Lymph node metastasis (No vs Yes)	*NA*	*NA*	*p* = 0.962	*p* = 0.516	*p* = 0.879	*p* = 0.892
Distant metastasis (No vs Yes)	*p* = 0.251	*NA*	*p* = 0.498	*p* = 0.889	*p* = 0.979	*p* = 0.791
Vascular invasion (No vs Yes)	*p* = 0.371	*p* = 0.551	*p* = 0.101	*p* = 0.524	*p* = 0.352	*p* = 0.428
Child-Pugh grade (A vs B or C)	*p* = 0.860	*p* = 0.192	*p* = 0.036*	*p* = 0.770	*p* = 0.867	*p* = 0.600
HBsAg (Negative vs Positive)	*p* = 0.034*	*p* = 0.487	*p* = 0.761	*p* = 0.961	*p* = 0.583	*p* = 0.273
Anti-HCV (Negative vs Positive)	*p* = 0.405	*p* = 0.300	*p* = 0.541	*p* = 0.937	*p* = 0.870	*p* = 0.504
Liver cirrhosis (Negative vs Positive)	*p* = 0.141	*p* = 0.626	*p* = 0.884	*p* = 0.651	*p* = 0.333	*p* = 0.298

* *p* value < 0.05 as statistically significant. NA: not applicable.

Table 4. Association of *MALAT1* genotypic frequencies with hepatocellular carcinoma laboratory findings.

Characteristic	AFP [a] (ng/mL)	AST [a] (IU/L)	ALT [a] (IU/L)	AST/ALT [a] ratio
**rs3200401**				
CC	3333.6 ± 1009.4	100.5 ± 10.7	233.9 ± 139.5	1.5 ± 0.1
CT + TT	2076.5 ± 942.5	151.7 ± 34.3	113.3 ± 22	1.4 ± 0.1
p value	0.136	0.268	0.822	0.624
p value [b]	0.280	0.040*	0.624	0.556
**rs619586**				
AA	3436.3 ± 884.3	120.1 ± 14.1	216.9 ± 111.5	1.5 ± 0.1
AG + GG	230.7 ± 76	104 ± 40.1	74.6 ± 10.8	1.4 ± 0.1
p value	0.116	0.085	0.946	0.190
p value [b]	0.214	0.479	0.507	0.529
**rs1194338**				
CC	3059.6 ± 1241.7	97.6 ± 12.7	312.1 ± 213.2	1.5 ± 0.1
CA + AA	2804.1 ± 904.3	132.9 ± 21.8	102.1 ± 14.4	1.4 ± 0.1
p value	0.962	0.344	0.375	0.803
p value [b]	0.680	0.158	0.261	0.532

Mann-Whitney U-test was used between two groups. Multiple linear regression was used to adjust for age, gender, cigarette smoking, and alcohol consumption. [a] Mean ± standard error. [b] Adjusted for age, gender, cigarette smoking, and alcohol consumption. AFP, alpha-fetoprotein; AST, aspartate aminotransferase; ALT, alanine aminotransferase.

Table S5. Odds ratio (OR) and 95% confidence interval (CI) of clinical status and *MALAT1* genotypic frequencies in hepatocellular carcinoma patients with hepatitis B virus infection (*n* = 167)

Variable	Genotypic frequencies		
	**rs3200401**	**rs619586**	**rs1194338**
Clinical Stage (Stage I/II vs Stage III/IV)	*p = 0.615*	*p = 0.695*	*p = 0.426*
Tumor size (≦T2 vs. > T2)	*p = 0.581*	*p = 0.542*	*p = 0.501*
Lymph node metastasis (No vs Yes)	*p = 0.616*	*p = 0.999*	*p = 0.396*
Distant metastasis (No vs Yes)	*p = 0.473*	*p = 0.877*	*p = 0.848*
Vascular invasion (No vs Yes)	*p = 0.550*	*p = 0.566*	*p = 0.228*
Child-Pugh grade (A vs B or C)	*p = 0.865*	*p = 0.086*	*p = 0.107*
Anti-HCV (Negative vs Positive)	*p = 0.978*	*p = 0.614*	*p = 0.951*
Liver cirrhosis (Negative vs Positive)	*p = 0.108*	*p = 0.284*	*p = 0.312*

Table 6. Odds ratio (OR) and 95% confidence interval (CI) of clinical status and *MALAT1* genotypic frequencies in hepatocellular carcinoma patients with hepatitis C virus infection (*n* = 178).

Variable	Genotypic frequencies		
	**rs3200401**	**rs619586**	**rs1194338**
Clinical Stage (Stage I/II vs Stage III/IV)	*p = 0.487*	*p = 0.594*	*p = 0.912*
Tumor size (≦T2 vs > T2)	*p = 0.849*	*p = 0.644*	*p = 0.750*
Lymph node metastasis (No vs Yes)	*NA*	*NA*	*p = 0.452*
Distant metastasis (No vs Yes)	*NA*	*NA*	*p = 0.452*
Vascular invasion (No vs Yes)	*p = 0.322*	*p = 0.170*	*p = 0.497*
Child-Pugh grade (A vs B or C)	*p = 0.649*	*p = 0.476*	*p = 0.998*
HBsAg (Negative vs Positive)	*p = 0.634*	*p = 0.554*	*p = 0.702*
Liver cirrhosis (Negative vs Positive)	*p = 0.435*	*p = 0.761*	*p = 0.189*

NA: not applicable.
